# Schizophrenia Genomics: Convergence on Synaptic Development, Adult Synaptic Plasticity, or Both?

**DOI:** 10.1016/j.biopsych.2021.10.018

**Published:** 2022-04-15

**Authors:** Jeremy Hall, Nicholas J. Bray

**Affiliations:** MRC Centre for Neuropsychiatric Genetics & Genomics, Division of Psychological Medicine & Clinical Neurosciences, Cardiff University, Cardiff, United Kingdom; Neuroscience & Mental Health Research Institute, Cardiff University, Cardiff, United Kingdom

**Keywords:** Genetics, Genomics, Neurodevelopment, Schizophrenia, Synapse, Synaptic plasticity

## Abstract

Large-scale genomic studies of schizophrenia have identified hundreds of genetic loci conferring risk to the disorder. This progress offers an important route toward defining the biological basis of the condition and potentially developing new treatments. In this review, we discuss insights from recent genome-wide association study, copy number variant, and exome sequencing analyses of schizophrenia, together with functional genomics data from the pre- and postnatal brain, in relation to synaptic development and function. These data provide strong support for the view that synaptic dysfunction within glutamatergic and GABAergic (gamma-aminobutyric acidergic) neurons of the cerebral cortex, hippocampus, and other limbic structures is a central component of schizophrenia pathophysiology. Implicated genes and functional genomic data suggest that disturbances in synaptic connectivity associated with susceptibility to schizophrenia begin in utero but continue throughout development, with some alleles conferring risk to the disorder through direct effects on synaptic function in adulthood. This model implies that novel interventions for schizophrenia could include broad preventive approaches aimed at enhancing synaptic health during development as well as more targeted treatments aimed at correcting synaptic function in affected adults.

The synapse is the principal means of neuronal communication and therefore central to all brain functions, with more than 160 trillion synapses estimated in the human cerebral cortex alone (1). Unsurprisingly, postulated disturbances in synaptic function and connectivity have figured prominently in neurobiological theories of schizophrenia (2, 3, 4, 5, 6, 7, 8, 9, 10), supported by neuropathological (11,12), neuropharmacological (13,14), and genomic (15) studies. Two recent landmark genomic studies of schizophrenia (16,17) provide further evidence for a synaptic component to the condition and bring into sharper focus some of the genes that are etiologically relevant. In the following review, we discuss these and other genomic insights into schizophrenia susceptibility in the context of gene expression and synaptic function in the developing and adult brain.

## Synaptic Genes and Genetic Risk for Schizophrenia

**Figure 1:**
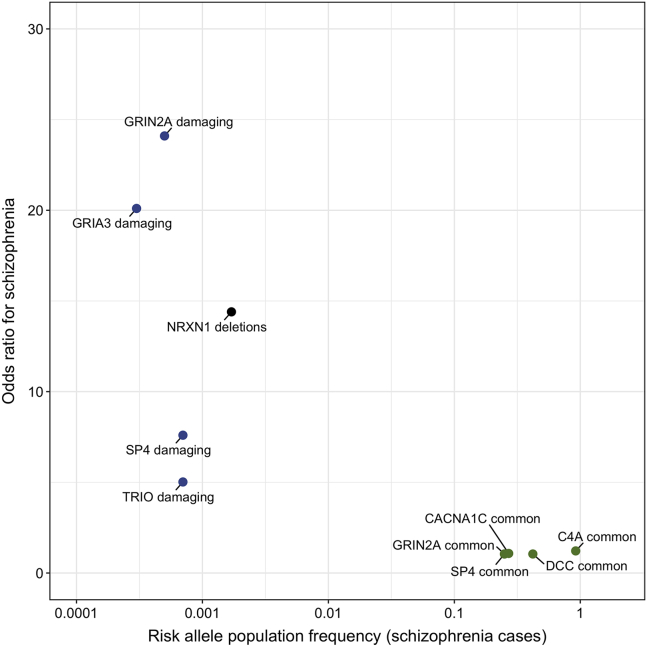
Estimated odds ratios for DNA variants at example synaptic gene loci associated with schizophrenia and their observed frequency in schizophrenia cases in the corresponding studies. Damaging variants (blue dots) encompass rare protein-truncating variants and missense (nonsynonymous) coding variants with an MPC (missense badness, polyphen-2, constraint) pathogenicity score >3, as identified in the recent exome sequencing study of the Schizophrenia Exome Meta-Analysis (SCHEMA) Consortium (17). Estimated schizophrenia odds ratio and population frequency of *NRXN1* deletions (black dot) are calculated from the copy number variant meta-analysis of Marshall *et al.* (20). Estimated schizophrenia odds ratios and population frequencies of common (>1% population frequency) DNA variants (green dots) are derived from the recent genome-wide association study of the Schizophrenia Working Group of the Psychiatric Genomics Consortium (16).

Advances in genotyping and sequencing technology combined with large sample sizes have led to the identification of robust genetic associations with schizophrenia over the past decade. These have revealed a complex polygenic architecture for the disorder, involving numerous common (>1% population frequency) genetic susceptibility variants of individually low penetrance as well as rarer genetic variants that can have stronger effects on schizophrenia risk (18) (Figure 1).

Genome-wide association studies (GWASs) test association between a trait and more than a million common genetic variants spanning the human genome. A great advantage of GWASs over earlier candidate gene association studies is that they are agnostic in design, requiring no prior knowledge of pathophysiology or of the genes that might be involved. However, this comes at a cost of a huge multiple-testing penalty (the accepted threshold for genome-wide significance in GWASs is *p* < 5 × 10^−8^), and therefore, very large sample sizes are required to confidently detect common risk variants of the effect sizes generally observed for schizophrenia (common variant odds ratios typically <1.1). The most recent schizophrenia GWAS of the international Psychiatric Genomics Consortium (PGC) meta-analyzed genotyping data from approximately 70,000 people with schizophrenia and approximately 240,000 unaffected control subjects, identifying more than 250 common genetic loci associated with the disorder at genome-wide significance (16). Although it is difficult to confidently identify the relevant genes at individual GWAS loci [owing to often broad regions of genetic association, the predominantly noncoding nature of common risk alleles, and the potential for long-range gene regulation (19)], genetic associations were found to be significantly enriched within genes belonging to Gene Ontology sets relating to the synapse, ion channels, or neurodevelopment (16).

Studies of rare genetic variation have also highlighted an important role for synaptic genes in schizophrenia susceptibility. Copy number variants (CNVs) are large deletions or duplications that can result in loss or gain of genic sequence. In the largest CNV study of schizophrenia to date (20), rare (<1% population frequency) deletions identified in schizophrenia were found to disproportionally affect genes relating to synaptic function, with the Gene Ontology term “synaptic” and the activity-regulated cytoskeleton protein complex [as defined by Kirov *et al.* (21)] ranked first for statistical significance and effect size, respectively. In addition, the meta-analysis identified 8 individual CNV loci associated with schizophrenia at genome-wide significance. While schizophrenia-associated CNVs at most of these loci encompass multiple genes, they also include deletions that specifically disrupt *NRXN1*, encoding the synaptic cell-adhesion protein neurexin-1, which have an estimated odds ratio of approximately 14 (20).

Exome sequencing allows identification of rare protein-damaging coding genetic variants at single-nucleotide resolution. Such analyses have highlighted an excess of loss-of-function and missense genetic variants in genes encoding synaptic proteins involved in the NMDA receptor complex and postsynaptic density as well as voltage-gated calcium channels in schizophrenia (17,22, 23, 24). In the recent large-scale study of the Schizophrenia Exome Meta-Analysis (SCHEMA) Consortium based on data from more than 24,000 schizophrenia cases and 97,000 control subjects, 10 genes were found to contain an exome-wide significant excess of ultrarare protein-damaging coding mutations in schizophrenia (17). These include a gene encoding an NMDA receptor subunit that is also implicated in common genetic risk for schizophrenia through GWAS fine-mapping (*GRIN2A*; estimated odds ratio for highly damaging coding variants ∼24.1), a gene encoding a glutamatergic AMPA receptor subunit (*GRIA3*; estimated odds ratio for highly damaging coding variants ∼20.1), and a gene encoding a synaptic voltage-gated calcium channel (*CACNA1G*; estimated odds ratio for highly damaging coding variants ∼4.25).

Thus, both common and rare variant genomic studies implicate genes that are directly involved in synaptic activity and plasticity in risk for schizophrenia (15, 16, 17). However, these synaptic processes are important in shaping neural circuits during development as well as for mature brain function (10). Gene set enrichment analyses, although implicating synaptic biology in schizophrenia, are currently limited by our incomplete understanding of gene function and do not necessarily inform as to the developmental timing of the risk mechanisms. In addition, and as we discuss later, several genes implicated in schizophrenia through genomic studies have reported roles in early synapse formation. This raises the question of at which stage(s) of development do synaptic disturbances confer susceptibility to the disorder.

## The Neurodevelopmental Hypothesis of Schizophrenia

One of the most influential theories of the etiology of schizophrenia, first expounded in the 1980s (25,26), holds that the condition has its fundamental origins in early (prenatal) brain development. A key source of evidence supporting the neurodevelopmental hypothesis of schizophrenia comes from epidemiological studies. A number of early-life insults have been shown to increase risk for the condition, including prenatal infections, obstetric complications, and maternal famine/nutritional deficiency during pregnancy (27). Large-scale longitudinal cohort studies have provided evidence that people who later develop schizophrenia have, on average, subtle impairments of cognition, behavior, and development in their childhood years relative to expectations (28,29). The presence of gross neuroanatomical deviations (e.g., ventricular enlargement) at schizophrenia onset and a general absence of markers of neurodegeneration are further cited as support for a neurodevelopmental model of the disorder (30).

The neurodevelopmental hypothesis acknowledges that notwithstanding the subtle premorbid deficits outlined above, the pre- or perinatal component of schizophrenia does not generally manifest as overt psychosis until late adolescence. Indeed, it is difficult to predict who will develop schizophrenia even when dealing with high-risk groups at the period of greatest risk of transition to the disorder (31). To account for the delayed manifestation of the illness, the classic neurodevelopmental model holds that the effects of early insults on the developing brain are only fully revealed when the brain matures in early adulthood (consequent on, for example, synaptic pruning and myelination of the frontal cortex). Support for this view is provided by animal studies showing that effects of neonatal hippocampal lesions on schizophrenia-relevant phenotypes, such as sensorimotor gating deficits and sensitivity to stress, can remain latent until maturity (32,33).

## Functional Genomic Support for an Early Neurodevelopmental Component to Schizophrenia

Functional genomic studies provide strong support for a prenatal component to schizophrenia (34). For example, Gulsuner *et al.* (35) found that genes containing damaging de novo mutations in the disorder were most significantly coexpressed in the frontal cortex during fetal development rather than during childhood and adolescence/adulthood. Similarly, Clifton *et al.* (36) found that common variant genetic association with schizophrenia was positively correlated with relative expression of the corresponding genes in the prefrontal cortex during the second trimester of gestation and early infancy. Moreover, common risk variants for schizophrenia are significantly overrepresented within genetic variants affecting DNA methylation (methylation quantitative trait loci) (37), gene expression (expression quantitative trait loci) (eQTL) (38,39), and gene splicing (splicing quantitative trait loci) (39) in the human fetal brain, indicating likely mechanisms by which they operate.

## Early Synaptic Connectivity and Genetic Risk for Schizophrenia

The establishment of synaptic connectivity in the human cerebral cortex begins in the second trimester of gestation, as differentiating neurons that have migrated to their target destination start to extend axons and dendrites. This is followed by a period of intense synaptogenesis during the third trimester of pregnancy that continues into early childhood (40, 41, 42). There is now good evidence that at least some of the genetic risk factors for schizophrenia operating in the fetal brain directly affect the establishment of synaptic connectivity. For example, *NRXN1*, which is most highly expressed in late gestation and early childhood (43) and is a target of schizophrenia-associated CNVs (20), appears to serve a role in synapse formation (44,45). Of the 10 genes found to harbor an exome-wide significant excess of rare damaging coding mutations in schizophrenia in the SCHEMA Consortium analysis (17), *TRIO* is known to play an important role in developmental neurite outgrowth (46), while *Setd1a* haploinsufficiency in mice has been reported to reduce axonal branching (47) and *SP4* knockdown to affect dendritic branching (48) during development. The recent PGC GWAS (16) included fine-mapping of schizophrenia-associated single nucleotide polymorphisms to several individual genes with known roles in neurite outgrowth and synapse formation, including *ZNF804A* (49), in which schizophrenia risk variation may act specifically during fetal brain development (50,51); *CNTN4* (52); *LRRC4B* (53); and *DCC* (54), the last encoding a Netrin-1 receptor known to interact with *TRIO* (46). In addition, studies that integrate schizophrenia GWAS data with eQTL/splicing quantitative trait loci operating in the human fetal brain provide evidence for altered prenatal regulation of several genes involved in neurite outgrowth in association with genetic risk for the condition, including *CNTN4* (39,52) and genes within the protocadherin alpha cluster (55,56). These findings suggest that synaptic disturbances conferring risk for schizophrenia begin in utero. However, the expression of several of these molecules persists throughout postnatal brain development (57), when they may additionally contribute to synaptic maturation in response to environmental stimuli (10) and adult synaptic function.

## Functional Genomic Support for Schizophrenia Risk Mechanisms Operating in the Adult Brain

Common genetic risk variants for schizophrenia are enriched for eQTL operating in the adult (58,59) as well as fetal (38,39) human brain, and many of the associated prenatal genetic effects on gene expression continue into adulthood (16,38,39,55,60). Schizophrenia associations are significantly overrepresented in genes with high relative specificity of expression in several regions of the adult human brain compared with other tissues, including the cerebral cortex, nucleus accumbens, hippocampus, amygdala, caudate, and cerebellum (16). Notably, in the adult human brain, schizophrenia single nucleotide polymorphism heritability is concentrated in regulatory genomic sites operating in neurons rather than glia (61,62). Indeed, schizophrenia associations are reported to be enriched within genes that show high specificity of expression in neurons from the cerebral cortex (pyramidal neurons and interneurons) and hippocampus (pyramidal and granule neurons) of the adult human brain (16) and additionally within genes with high specificity of expression in medium spiny neurons of the striatum in the more extensive single-cell datasets from adult mouse brain (16,63). These data suggest that genomic risk for schizophrenia is partly determined by neuron-specific processes operating in the mature brain, of which synaptic plasticity is a prime example.

## Adult Synaptic Plasticity and Genetic Risk for Schizophrenia

Synaptic plasticity is the property by which neurons modulate the strength of synaptic transmission and connectivity in response to activity and is thought to be the biological basis of associative learning and memory (64,65), abnormalities in which have been implicated in schizophrenia symptomatology (7,8). As discussed in an earlier review (15), findings from rare variant studies of schizophrenia show significant convergence on molecular pathways involved in synaptic plasticity. Both the glutamatergic NMDA receptor complex and voltage-gated calcium channels, which are strongly implicated in genetic risk for schizophrenia (15), play a central role in synaptic plasticity by allowing entry of Ca^2+^ into postsynaptic dendrites (66,67). This in turn activates second messenger systems that result in changes in synaptic efficiency through incorporation of glutamatergic AMPA receptors into the postsynaptic membrane and changes in the size and shape of dendritic spines.

The recent schizophrenia GWAS of the PGC (16) and the exome sequencing analysis of the SCHEMA Consortium (17) provide further evidence for the involvement of synaptic plasticity and adult memory processes in risk for schizophrenia. Indeed, *GRIN2A* and *SP4*, 2 genes implicated in schizophrenia susceptibility through both fine-mapping of GWAS loci (16) and exome sequencing (17), have direct roles in NMDA receptor function and associative memory. *GRIN2A* encodes the GluN2A subunit of the NMDA receptor. Unlike the other major GLuN2 subunit, GLuN2B, GluN2A is expressed predominantly postnatally, reaching maximal expression in adolescence (57,68). Prominent GluN2A expression is observed in the hippocampus (68,69), and hippocampal memory deficits are observed after adult GluN2A antagonism (70) and *Grin2a* knockout (71) in rodents. *SP4* encodes a transcription factor known to regulate NMDA receptor subunit expression (72,73). Although *SP4* expression is highest in the prenatal brain (57), it appears to play an important role in the adult hippocampus, with *Sp4* hypomorphic mice displaying deficits in hippocampal learning that could be rescued by restoration of *Sp4* function (74). Other genes with known roles in adult synaptic function implicated in schizophrenia by the SCHEMA exome sequencing analysis (17) are *GRIA3*, encoding the AMPA receptor subunit 3 (75), and *CACNA1G*, encoding the Cav3.1 channel (76,77), while such genes prioritized by fine-mapping schizophrenia-associated loci in the recent PGC3 GWAS (16) include *GRM1* (78), *GABBR2* (79), *CLCN3* (80), and *CACNA1C* (81). Recent transcriptome-wide association studies of schizophrenia based on eQTL identified in adult human brain provide evidence for altered expression of several synaptic plasticity genes in association with genetic risk for schizophrenia, including *CLCN3* (80), *GABRA2* (82), and *LRP8* (83) in the adult frontal cortex (60) and *GRM3* (84) and *CACNA1C* (81) in the adult dentate gyrus (85).

Schizophrenia-associated genes encoding molecules involved in neurite and synaptic development may also exert an effect on synaptic activity and remodeling in the adult human brain. For example, *NRXN1* is reported to increase Ca^2+^ influx through the NMDA receptor (86) and its splicing to affect the stability of hippocampal fear memories (87), while *TRIO* has been shown to support glutamatergic transmission and long-term potentiation in rodent hippocampal slice cultures (88). We believe that a systematic assessment of the role of well-supported schizophrenia susceptibility genes in both synaptic development and adult synaptic function is now warranted, with genes implicated through exome sequencing and fine-mapping of nonsynonymous coding variants prioritized and with due attention to the specific RNA transcripts affected by genetic risk variation. Here, use of age-specific gene knockout strategies and advanced in vitro models (e.g., brain organoids) are likely to be informative.

Although functional genomic data provide evidence for adult neuronal mechanisms in schizophrenia susceptibility, genetic enrichment studies based on gene expression in the postmortem brain might underestimate the role of schizophrenia risk genes in adult synaptic plasticity. This is because activity-related changes in gene expression, arising within specific neuronal circuits at particular points in time, are unlikely to be reliably captured in postmortem tissues. This contrasts with early brain development, when large gene expression programs are playing out en masse. The integration of human genomic findings with data from in vivo and in vitro model systems provides one means of assessing the role of activity-dependent gene expression in schizophrenia susceptibility. For example, Clifton *et al.* (89) found that genes with increased expression in the CA1 region of the rodent hippocampus after specific forms of associative learning were enriched for genes affected by CNVs in schizophrenia. Similarly, Roussos *et al.* (90) reported enrichment of common genetic risk variants for schizophrenia within a transcriptional gene network associated with depolarization of neurons derived from human induced pluripotent stem cells. Understanding how schizophrenia risk variation affects processes involved in adult synaptic plasticity is a key ongoing research question, which may have direct relevance to the development of new treatments.

## Synaptic Dysfunction as Both a Neurodevelopmental Antecedent and an Ongoing Risk Mechanism for Schizophrenia

The aforementioned suggests a model in which genetic risk for schizophrenia operates not only on the formation and maintenance of synaptic networks during brain development but also through direct effects on synaptic function and plasticity in the adult brain. This is supported by the findings of a recent study indicating that pronounced enrichment of common genetic risk variation for schizophrenia was found in human brain gene coexpression modules involved in synaptic transmission and neuronal excitability that are maintained, or increased, in expression from birth into adulthood (91). Such a model is also consistent with key aspects of several other neurobiological theories of schizophrenia based on epidemiological, neuropathological, neuroimaging, neuropharmacological, and cognitive findings (2, 3, 4, 5, 6, 7, 8, 9, 10).

**Figure 2:**
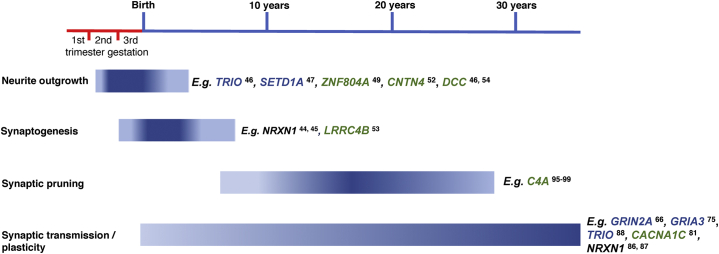
Developmental timing of synaptic processes in the human frontal cortex of potential relevance to schizophrenia (40, 41, 42,94). Examples of genes implicated in schizophrenia susceptibility that have been reported to serve a role in these processes are provided (references in superscript). Genes in blue font are implicated in schizophrenia risk through exome sequencing (17); genes in green font are implicated through fine-mapping of genome-wide association study risk loci (16); *NRXN1* is implicated through copy number variation disrupting this gene (20). Note that individual genes can be in involved in multiple synaptic and other biological processes.

In keeping with the classic neurodevelopmental hypothesis (25,26), genetic and functional genomic studies support the notion that schizophrenia pathogenesis begins in utero. As we have noted, several of the genes that are strongly implicated in schizophrenia risk through exome sequencing and fine-mapping of GWAS loci have known roles in neurite outgrowth and synaptogenesis, suggesting that disturbances in the initial formation of synaptic connectivity are important for later expression of the condition. From birth, synaptic networks are shaped and refined by activity and experience, with up to half of synapses being eliminated through synaptic pruning and others being strengthened through synaptic maturation (41). Synaptic activity through the NMDA receptor appears to be essential for this postnatal development and refinement of excitatory connections (92,93). Differences in NMDA receptor activity through schizophrenia-associated genetic effects on *GRIN2A* is therefore likely to alter the maturation of relevant glutamatergic synapses and have a direct impact on synaptic plasticity throughout postnatal life. Developmental synaptic pruning, which in the frontal cortex extends into early adulthood (40,94), is a prime candidate for exposing and exacerbating compromised neuronal networks and has long been postulated as a schizophrenia risk mechanism in itself (2,4). Indeed, several groups have reported increased synaptic elimination with higher expression of complement C4/C4A (95, 96, 97, 98, 99), which is elevated in the human brain in association with schizophrenia risk variation at the *C4A* gene locus (38,99). Other gene variants may increase the risk for schizophrenia by affecting neuronal function primarily in the mature brain. For example, genes within the schizophrenia-associated Gene Ontology term of “voltage-gated calcium channel activity” display highest expression in the dorsolateral prefrontal cortex in adolescence and early adulthood (100). Figure 2 shows the developmental timing of various synaptic processes that appear relevant to schizophrenia genetic risk, with examples of implicated genes that have been reported to serve a role in these processes.

Convergent genetic evidence for *GRIN2A* as a schizophrenia susceptibility gene provides strong support for long-held (hypo-) glutamatergic theories of the disorder that were based on the observed induction of positive, negative, and cognitive symptoms in healthy adults after NMDA receptor antagonism (13,101). Although common genetic risk variation for schizophrenia is enriched within genes expressed across multiple regions of the adult human brain, there appears to be pronounced signal in genes with higher specificity for expression in the cerebral cortex and limbic structures (nucleus accumbens, amygdala, and hippocampus) (16). Synaptic disturbances in these regions of the adolescent and adult brain are likely to affect working memory, executive function, associative learning, and predictive learning processes that are hypothesized to be central to cognitive, motivational, and psychotic symptoms of the disorder (7, 8, 9,102, 103, 104). The effects of genetic risk variants on adult synaptic plasticity may be particularly marked in the context of preexisting neurodevelopmental alterations in brain circuitry.

Risk for schizophrenia is not solely determined by genetics and, as noted above, a number of pre- and perinatal environmental exposures are also associated with later development of the condition (27). Both prenatal nutritional deficiency and obstetric complications have been shown to have an impact on the developing hippocampus, with later effects on cognition and behavior (105,106). Postnatal maturation and shaping of neuronal circuits is governed by activity and experience, mediated through synaptic plasticity mechanisms, with environmental enrichment found to increase dendritic spine density (107,108). Conversely, psychological stress, a precipitating factor for various neuropsychiatric disorders, is associated with volume reductions in the prefrontal cortex and hippocampus and has been reported to reduce dendritic spine density in these areas in rodents (109), while repeated exposure to Δ^9^-tetrahydrocannabinol, the main psychoactive ingredient in cannabis, might have an impact on schizophrenia risk through reported effects on hippocampal dendritic spine density and glutamate receptor subunit expression (110). There is evidence that some of these environmental factors have more potent effects on schizophrenia susceptibility in individuals of high genetic risk for the disorder (i.e., gene-environment interaction) (111,112).

## Synaptic Dysfunction Across the Neuropsychiatric Spectrum

Genetic evidence for disturbances in synaptic development and function is not confined to schizophrenia. For example, rare variants affecting genes involved in synaptic connectivity, including *NRXN1*, have been strongly implicated in autism (113), and common variants in or near genes encoding voltage-gated calcium channels (e.g., *CACNA1C*) are also associated with bipolar disorder (114). Indeed, a GWAS meta-analysis across 8 neuropsychiatric conditions (anorexia nervosa, attention-deficit/hyperactivity disorder, autism, bipolar disorder, major depression, obsessive-compulsive disorder, schizophrenia, and Tourette syndrome) found pleiotropic (shared) loci to be enriched for genes involved in glutamate receptor signaling and voltage-gated calcium channel complexes as well as genes involved in neural development (115). However, compared with schizophrenia, autism is associated with a higher burden of rare damaging variants (116) [which may additionally affect earlier neurodevelopment processes, such as cell proliferation (117)], while bipolar disorder has a lower burden of rare CNVs (118, 119, 120). As argued by Owen *et al.* (121,122), evidence suggests that neuropsychiatric disorders reflect a gradient of early neurodevelopmental disturbance, with greater prenatal impacts in childhood-onset conditions, such as autism, than in schizophrenia, and with diagnoses such as bipolar disorder and major depression associated with less neurodevelopmental disruption still [although common risk variants for these latter conditions may still operate, albeit more subtly, in the prenatal brain (38,55,115,123,124)]. The extent of neurodevelopmental disturbance is also likely to explain some of the clinical heterogeneity in schizophrenia. For example, in the initial report of a genome-wide significant association between loss-of-function variants in *SETD1A* and schizophrenia (116), 7 of 10 people with schizophrenia carrying these mutations also had learning difficulties. A corollary is that in cases of schizophrenia when cognition is relatively spared, there may be less early developmental disruption. Improved linkage between genetic and clinical data might help refine the biological processes contributing to the diverse symptomatology and course of the disorder.

## Implications for Treatment

The above model suggests two strategies for reducing the incidence of schizophrenia and its severity: first, a broad preventive approach aimed at maximizing synaptic integrity during brain development, and second, focused treatments aimed at ameliorating deficits in synaptic function after the onset of psychosis. Given that prenatal brain development appears to be an important variable in later risk for schizophrenia, universal strategies for optimizing nutrition and maternal care during pregnancy could, if defined appropriately, be a cost-effective and feasible means of reducing the incidence of the disorder (125, 126, 127). In people who have already experienced a psychotic episode, targeted strategies based on restoring cortical and hippocampal synaptic function may be effective in treating cognitive and other symptoms of the condition. Indeed, there is encouraging evidence from randomized, placebo-controlled trials that augmentation of typical antipsychotic treatment with agonists of the glycine site of the NMDA receptor reduces both positive and negative symptoms in patients with schizophrenia (128). Current large-scale genomic studies of schizophrenia may suggest further targets for drug development (129).

## Conclusions

Both common and rare variant genomic analyses provide strong support for a synaptic component to schizophrenia etiology, consistent with a number of long-standing neurobiological theories of the disorder. Implicated genes and functional genomic data suggest that synaptic disturbances begin in utero but continue throughout development, with some alleles conferring risk for schizophrenia through direct effects on synaptic function in adulthood. However, a more precise understanding of synaptic processes contributing to the disorder will require elucidation of the particular gene transcripts that are affected by genetic risk variation and the cellular specificity of these effects, together with a systematic investigation of their role in synaptic development and function in relevant cell types and models. With advances in RNA sequencing, single-cell technologies, in vitro models, and genome editing, such investigations are already becoming possible. It is hoped that the insights provided will pave the way for improved treatments for schizophrenia, which might even include strategies to prevent its onset.
